# Resequencing of the Red Alga Cyanidioschyzon merolae: Strain Differences and Mitochondrial Sequence Corrections

**DOI:** 10.1128/MRA.00285-20

**Published:** 2020-06-11

**Authors:** Naoki Sato, Takashi Moriyama, Natsumi Mori-Moriyama

**Affiliations:** a Department of Life Sciences, Graduate School of Arts and Sciences, University of Tokyo, Tokyo, Japan; Indiana University, Bloomington

## Abstract

Two laboratory strains of the red alga Cyanidioschyzon merolae 10D were resequenced. We found some strain differences in the nuclear and chloroplast genomes. We also identified corrections of the mitochondrial genome sequence.

## ANNOUNCEMENT

Cyanidioschyzon merolae is a unicellular red alga that was sequenced in 2004 and updated in 2007 (1, 2). The nuclear genome was 16.4 Mbp with 54.8% GC content. We noted significant growth differences in two laboratory strains, GT (glycerol tolerant) and 10D-T (from the Tanaka laboratory), both of which are descendants of the originally sequenced strain 10D, which is no longer available. We kept GT for 10 years with glycerol supplementation (3); it grows better than 10D-T under standard growth conditions (Table 1). The cell sizes were also significantly different; namely, the length and width of GT cells were 1.18 and 1.30 times larger than those of 10D-T cells, respectively (average, 231 and 320 cells, respectively; *P* < 10^−51^).

**TABLE 1:** Genome data for the two strains of Cyanidioschyzon merolae

Characteristic	Data for strain:	
	10D-T	GT
Accession no. of raw reads	DRX207478	DRX207477
Accession no. of draft genome	BLLC00000000	BLLD00000000
Accession no. of mitochondrial genome	LC519602	Identical to 10D-T
No. of read pairs	14,075,034	13,256,078
No. of contigs	2,672	2,649
*N*_50_ contig size (bp)	17,362	17,150
GC content (%)	54.3	54.9
Doubling time (h)^*a*^	14.6 ± 0.2	13.0 ± 0.3

a Average ± SD of three measurements; *P *= 0.003.

*C. merolae* cells were grown in 2× Allen medium with 1.0% CO_2_ aeration at 42°C (3). DNA mainly from the nuclear genome (but still containing chloroplast and mitochondrial DNA) was purified by CsCl density gradient centrifugation (4). Library construction and sequencing were provided as a custom service of Eurofins Genomics K. K. (Tokyo, Japan). Genomic DNA samples were sheared to 300-bp fragments by sonication with an M220 sonicator (Covaris, MA). The resulting DNA fragments were processed for adaptor ligation and amplification to generate DNA libraries. The prepared libraries were subjected to paired-end 2 × 125-bp sequencing (about 190× coverage) on the HiSeq 2500 platform (Illumina, San Diego, CA).

Whole-genome assemblies were constructed with MIRA version 4.0.2 with default settings (5). Mapping of the reads onto the published sequence was performed with inGap version 2.8.2 with default settings (6). The results revealed various differences between the two strains, as well as differences from the original, published sequence. In the nuclear genome, the two strains were found to have 288 single nucleotide polymorphisms (SNPs) and 81 indels in common with the published sequence. There were 118 SNPs and 37 indels that were found only in 10D-T, and 67 SNPs and 10 indels only in GT. In chromosome 15, the deletion of G at 592,904 (within the gene encoding CMO228C or Tic236) that was reported previously (7) was confirmed.

In the chloroplast genome (GenBank accession number AB002583), a 12-bp tandem repeat (nucleotide positions 39384 to 39395) in the *gltB* gene (encoding glutamate synthase large subunit) was missing in 10D-T, which could account for the growth difference. Nevertheless, this 4-amino-acid deletion (599 SESK 602) is located in a poorly conserved loop region and did not affect nitrogen assimilation activity as far as we measured. Two additional deletions (nucleotide positions 70213 to 70223 and 80473 to 80484) of repeats were detected in the chloroplast genome of 10D-T, but these do not affect gene structure. The chloroplast genome of GT was identical to the reported one.

In addition, we found 33 SNPs and 13 indels with respect to our previously published mitochondrial sequence (GenBank accession number D89861) (8) which are conserved in both GT and 10D-T. Accordingly, frameshift changes were found in the *nad5*, *rps14*, and *rpl14* genes. Some base changes were also found in the *rrnS* and *rrnL* genes.

### Data availability.

The draft genome sequences of the two Cyanidioschyzon merolae strains were deposited in DDBJ/EMBL/GenBank under the accession numbers listed in Table 1. The versions described in this paper are the first versions.
